# Effect of Expertise on Boundary Extension in Approach Sequences

**DOI:** 10.1177/2041669517723652

**Published:** 2017-09-13

**Authors:** Emmanuelle Ménétrier, André Didierjean, Frédérique Robin

**Affiliations:** Université d’Angers, Angers, France; Université de Bourgogne-Franche-Comté, Besançon, France; 27045 Université de Nantes, Nantes, France

**Keywords:** scene perception, boundary extension, expertise, car driving

## Abstract

In a constantly changing environment, one of the conditions for adaptation is based on the visual system’s ability to realize predictions. In this context, a question that arises is the evolution of the processes allowing anticipation with regard to the acquisition of knowledge relative to specific situations. We sought to study this question by focusing on boundary extension, the tendency to overestimate the scope of a previously perceived scene. We presented to novice, beginner, and expert car drivers road scenes in the form of approach sequences constituting very briefly displayed photographs (i.e., 250 milliseconds each), in order to determine the effect of expertise at an early stage of scene perception. After three presentations, participants had to judge whether a fourth photograph was the same, closer up, or further away than the third one. When experts and beginners showed a classical boundary extension effect, novices presented no directional memory distortion. Different hypotheses are discussed.

In a constantly changing environment, one of the conditions for adaptation is based on the visual system’s (VS) ability to realize predictions. Some of these predictions deal with the likely future trajectory followed by a moving object, or the probable evolution of a dynamic visual scene in which the observer is embedded, a phenomenon called representational momentum (RM; Freyd & Finke, 1984; e.g., Hubbard, 2015; Didierjean, Ferrari, & Blättler, 2014, for reviews). However, the predictions realized by the VS also concern areas of the environment just outside the observer’s visual field. This phenomenon is known as boundary extension (BE; Intraub & Richardson, 1989; e.g., Intraub, 2012, for a review) and refers to the tendency to overestimate the scope of a previously perceived scene, with the observer’s memory including information relative to the information likely to have been just outside the original scene’s boundaries.

While this phenomenon is often described as an error relative to the content of a previously perceived scene, it nevertheless presents an important adaptive value; indeed, it enables the VS to realize good predictions relative to the information present just beyond the edges of a view (e.g., Intraub & Dickinson, 2008). Moreover, BE is observed both with paradigms that simulate visual scanning—that is, with photographs presented for durations commensurate with an eye fixation (250 milliseconds) and with retention intervals imitating a saccade (42 milliseconds)—and with saccade-contingent paradigms in which the phenomenon is tested after an actual saccade (e.g., Intraub & Dickinson, 2008). Theoretically, this observation is significant as it suggests that anticipating the upcoming layout plays a role in the process of visual integration. It also suggests that BE participates in coherent and continuous representations of the surrounding world (e.g., Intraub & Dickinson, 2008), for instance, by priming the visual perception of the upcoming layout (Gottesman, 2011). To account for the rapid deployment of BE, Intraub and Dickinson (2008) suggested that the phenomenon should not result from an *ad hoc* extrapolation process, but may be deployed during scene understanding, the detection of layout automatically activating a larger spatial framework. Thus, this phenomenon has been observed in various situations, such as situations in which one can expect good memory (e.g., with low memory loads, cf., Munger, Owens, & Conway, 2005), during the life span (Seamon, Schleger, Heister, Landau, & Blumenthal, 2002), or in the haptic modality. In this last case, BE has been observed in equivalent proportions between blindfolded-sighted subjects and a blind-and-deaf observer, who was also considered as a “haptic expert” (Intraub, 2004). The fact that BE has been generalized to other sensory modalities suggests that anticipating the upcoming layout is a fundamental aspect of scene representation. More recently, Intraub, Morelli, and Gagnier (2015) even suggested that observation of BE in different sensory modalities underlies a unitary scene representation.

These observations led Intraub and her colleagues to develop an alternative model to the traditional visual-cognitive model of scene perception, the *Multisource model* (e.g., Intraub, 2012). Contrary to the traditional model that argues that scene perception relies on a single source of information, that is, the visual input, the *Multisource model* postulates that scene perception relies on different sources of information, with spatial information at its core. This model is thus close to grounded cognition theories (e.g., Barsalou, 2008) insofar as it considers that the observer is part of the scene he or she perceives. During perception, the egocentric framework generated by spatial information is “filled in” by other sources of information, such as visual and amodal information, the function of the latter being to continue the textures and surfaces of the scene just beyond the edges of the perceived view. Moreover, during the first fixation on a view, the rapid identification of the semantic category (i.e., gist) to which the perceived scene belongs (e.g., Potter, 1976) activates conceptual and contextual knowledge that generate expectations relative to its surroundings (Intraub, 2012). Thus, the extrapolation process would occur during scene perception (e.g., Intraub & Dickinson, 2008) by continuing the surfaces and textures of the scene just beyond the edges of the view in an amodal form (i.e., abstract). When the stimulus disappears, the observer encounters difficulty in discriminating the extrapolated information (abstract) from the visual information (concrete), resulting in spatial layout extrapolation. In this regard, BE is apprehended as a source monitoring error (Johnson, Hashtroudi, & Lindsay, 1993), in which a part of the internally driven information is combined with external information.

According to the *Multisource model* (Intraub, 2012), top-down expectations derived from conceptual and contextual knowledge would play a central role in spatial layout extrapolation. While the literature stresses the importance of knowledge relative to the gist of the perceived scene in the BE process, a question that arises is whether spatial layout extrapolation is modulated by the knowledge the observer has of the perceived scenes. The cognitive expertise paradigm seems relevant to address this question. Indeed, it permits variation in the amount of information available in LTM by contrasting novices and experts in a particular field. It is well known that expert knowledge modifies the perception of scenes depicting the perceiver’s field of expertise, experts processing these scenes more effectively than novices (e.g., Didierjean & Gobet, 2008, for a review). This cognitive advantage manifests itself, for instance, with a larger visual span (Reingold, Charness, Pomplun, & Stampe, 2001), the activation of automatic and parallel encoding procedures (Reingold, Charness, Schultetus, & Stampe, 2001), or the tendency to anticipate more than novices the probable evolution of the perceived situations (e.g., Didierjean & Marmèche, 2005).

In this study, we chose to compare the performances of novices and experts in the field of car driving. In most domains, age covaries with expertise. In addition to the two groups of subjects, we thus tested a third group of beginners belonging to the same novice age class. If we obtain an effect of expertise on BE, this group will enable us to determine whether the observed effect results from an effect of age or from an effect of the amount of information available in LTM.^1^ Moreover, as semantic knowledge related to the perceived scene is activated quickly after scene apparition (e.g., Davenport & Potter, 2004), we chose to test the effect of expertise at an early stage of scene perception by presenting road scenes for short presentation durations (250 milliseconds). To increase the ecological value of our stimuli, we decided to present the scenes in the form of approach sequences, a choice motivated by the fact that these sequences transmit the dynamic information acquired during real driving situations better than the same stimuli presented in a static form. Indeed, expert knowledge is often highly contextualized and influences behavior only in very ecological situations (e.g., Didierjean et al., 2014). Consequently, we used an adapted version of the camera distance paradigm (CDP; e.g., Intraub & Dickinson, 2008) developed by Munger et al. (2005) to determine whether BE and RM are related phenomena. In Munger et al.’s experiment, photographs were presented not in isolation, as in the classical version of the paradigm, but with sequences designed in such a way as to elicit RM by depicting an induced approach motion of the self. These sequences involved the display of three photographs that remained on screen very briefly. Each photograph was displayed for 250 milliseconds, followed by the presentation of a blank screen for 250 milliseconds (Figure 1). Immediately after the end of the sequence, a fourth photograph appeared and the observer was asked to judge whether this photograph was closer up, further away, or strictly the same as the last photograph in the sequence. The authors found not an RM effect (which would have manifested itself by remembering the last scene of the sequence as a closer up view) but rather a BE effect, leading them to suggest that gist information within a scene is processed before movement. They also observed variations of BE patterns on approach sequences as a function of individual differences in BE ratings obtained with single photographs. Indeed, the participants who extended boundaries with single photographs showed a trend toward less BE with approach sequences, whereas participants who did not extend boundaries (i.e., boundary restriction [the effect opposite to BE] or no directional distortion) of photographs presented in isolation showed BE with approach sequences. These observations suggest that BE is modulated by individual differences. In this sense, one can wonder whether differences in expert knowledge may be a factor of variation in spatial layout extrapolation.

**Figure 1.**
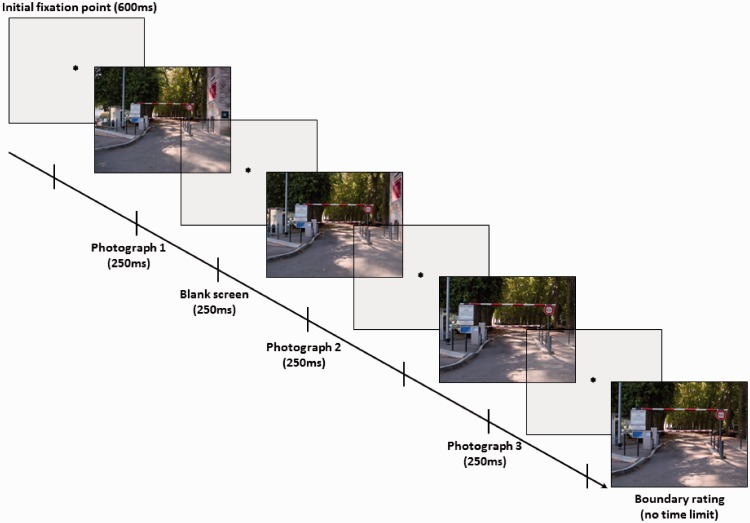
Depiction of a trial. Each trial was initiated by a 600-millisecond central fixation point before the approach sequence appeared on screen. The sequences constituted three briefly presented photographs, and participants had to compare the scope of a fourth picture to the third.

Our first objective is to replicate the observations realized by Munger et al. (2005). According to these authors, BE seems to occur before RM in this “competitive” situation between both effects, and we expect a BE phenomenon in all expertise levels. Our second objective is to test how BE is modulated by expert knowledge. Indeed, (a) as stated by the *Multisource model* (Intraub, 2012) which postulates that knowledge plays a central role in spatial layout extrapolation, and (b) as a result of the important amount of knowledge available in experts’ LTM (e.g., Didierjean & Gobet, 2008), we expect greater BE first in experts and then in beginners.

## Method

### Participants

Fifty-six subjects took part in the experiment and were divided into two groups according to their car driving expertise level:

‐ 20 novices (mean age = 19; *SD* = 1 year 4 months) who were students at the University of Franche-Comté (France) and who had never taken driving lessons.
‐ 20 beginners (mean age = 21; *SD* = 2 years) who were students at the University of Franche-Comté (France) and had had their driving licences for 2 years on average (mean = 23 months; *SD* = 18 months).
‐ 16 experts who were driving instructors (mean age = 39; *SD* = 9 years 1 month), and who declared that they had been giving driving lessons for 10 years on average (*SD* = 8 years). The choice of car driving instructors was motivated by the fact that they spend a lot of time on the road.

All participants had normal or corrected-to-normal vision. None was informed of the objectives of the experiment, and all of them gave their consent verbally.

### Materials

The experiment was presented on a 15-inch-screen MacBook Pro computer and was run by Psyscope software. During the experiment, participants were seated approximately 50 cm from the computer screen.

### Stimuli

Ten approach sequences constituting three photographs were created for this experiment. Each photograph depicted road scenes perceived from the driver’s viewpoint and began with a wide-angle version of the scene before finishing with a close-up version of the same scene. The second photograph (medium version) was realized by cropping the wide-angle version by approximately 30%, and the third photograph of the sequence (close-up version) was realized by cropping the medium version by about 30%. Each photograph was screen-centered and measured 14 × 10 cm. An additional sequence was presented during the familiarization phase.

### Procedure

We used an adapted version of the CDP to approach sequences developed by Munger et al. (2005). In this version of the paradigm, each of the photographs constituting the sequence was displayed very briefly, for 250 milliseconds, and the ISIs also lasted 250 milliseconds (cf. Figure 1). Two hundred and fifty milliseconds after the disappearance of the last photograph of the sequence, a fourth photo appeared on screen. The subject’s task was to compare the scope of the fourth stimulus to that of the third, by indicating on a 5-point scale whether this fourth photograph was strictly the same (0 value of the scale), a closer up version (a little: −1; much: −2), or a wider angle version (a little: 1; much: 2) of the third photograph. As in Munger et al.’s (2005) procedure, which did not use distractors (i.e., closer up and wider angle versions of the third photograph during boundary rating), the test photograph was always strictly the same as the third photograph and stayed on screen until the subject validated his or her answer (e.g., Gagnier & Intraub, 2012, for a version of the CDP without distractors). BE occurred when this photograph was judged to be closer up. Each sequence was preceded by a central fixation point of 600 milliseconds. Participants were exposed to the familiarization phase before individually completing 10 trials in a random order.

## Results

**Figure 2.**
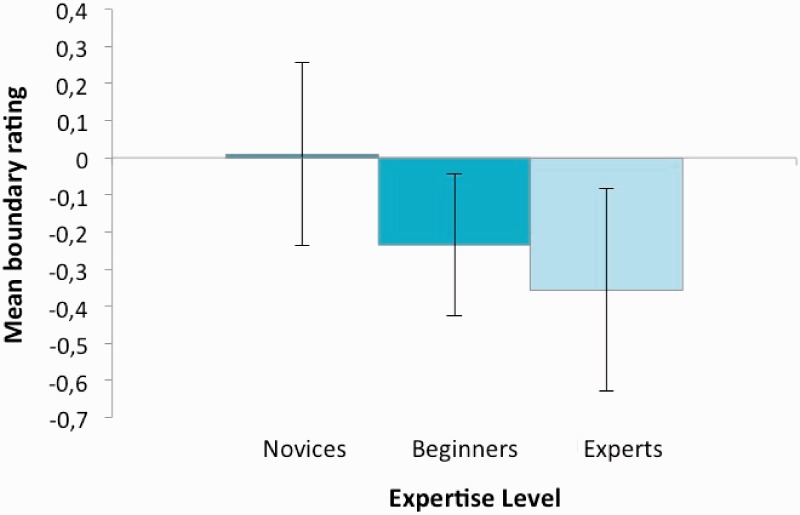
Mean boundary ratings observed as a function of expertise level. Error bars represent the confidence intervals (.95). Significant negative values indicate boundary extension.

Figure 2 represents the mean boundary ratings observed for each expertise level. To determine whether a memory distortion occurred, we computed the .95 confidence intervals (represented by error bars). If the mean boundary rating does not differ significantly from zero, no directional memory distortion has occurred. However, a significant negative value indicates that the observers’ representation included more background than the original picture, suggesting a BE effect. Conversely, a significant positive value shows that the observers’ memory included less background, demonstrating a boundary restriction effect. The mean boundary ratings observed for each expertise level are presented in Figure 2. While results indicate no directional distortion in novices, they reveal a BE effect in beginners and experts.

An ANOVA computed on subjects’ mean boundary ratings with Expertise as a between-subjects factor indicated an effect of expertise, *F*(2, 53) = 4.99, *p* = .01, *η*^2^^ ^= .16. Planned comparisons computed on novices’ and beginners’ mean boundary ratings indicated an effect of Expertise, *t*(38) = 2.09, *p* < .05*, d* = .66. Besides, the comparison of beginners’ and experts’ mean boundary ratings permitted us to ensure that the effect observed was not due to age, a factor that covariates with expertise. The results showed equivalent mean boundary ratings between these two groups of subjects, *t*(34) = 0.926, *ns*.

## Discussion

The principal objective of this study was to test the effect of expert knowledge on spatial layout extrapolation. We proposed a BE task during which road scenes were presented in the form of approach sequences to car drivers with different levels of expertise: novices, beginners, and experts. The results of the present study partially corroborate Munger et al.’s (2005) findings as they reveal a prevalence of BE and an absence of an RM-like effect. Moreover, BE appeared to be sensitive to expert knowledge with beginners and experts extrapolating spatial layout, whereas no directional memory distortion was observed in novices. These results indicate that expert knowledge is a source of expectations likely to modulate BE. Analogous to Munger et al.’s (2005) findings, one possible explanation is that beginners and experts may have incorporated a part of the larger spatial context perceived in the wide-angle version of the scene into their memory in equivalent proportions. This may have resulted in a source monitoring error (Johnson et al., 1993) in which the observers were unable to discriminate the contextual information acquired at the beginning of the approach sequence from the visual information actually depicted in the last photograph of the sequence. Given the contingencies of the BE task presented here, this interpretation differs slightly from the explanation traditionally provided by the *Multisource model*, which postulates that source monitoring errors causing BE result from a difficulty in discriminating external (i.e., visual and concrete) information from internally driven information (i.e., extrapolated information, which is abstract in nature). These observations suggest that BE is explained by a source-monitoring error which, depending on the tasks or the situations, may involve confusion between external and internal information in some cases and in others, as in the present study, a confusion between two external (i.e., concrete) sources of information.

The absence of directional memory distortion in novices can be linked to the observations realized by Blättler, Ferrari, Didierjean, and Marmèche (2011) in RM. In a study that aimed to examine the effect of expertise on RM, the authors proposed a classical RM task during which they presented simulated aircraft landing scenes to novices and experts (i.e., expert pilots from the French Air Force). While experts showed a classical RM effect, novices surprisingly showed no memory distortion. The authors interpreted this result as reflecting the weight of specific knowledge in motion extrapolation. They argued that while RM is a particularly robust phenomenon (Hubbard, 2015) that has been observed in many different situations (but see Kerzel, 2003), in the vast majority of studies, the observers were not actually real novices relative to the scenes presented. In their experiment, which used visual simulations based on synthesized images of aircraft landing scenes seen from the pilot’s viewpoint, novices were “true” novices and the RM effect was not observed. However, in our study, the accurate memory observed in novices can hardly be interpreted as a lack of semantic knowledge relative to road scenes as these scenes are familiar to novices, at least as passengers (e.g., Jordan & Hunsinger, 2008). As Ménétrier and Didierjean (2013) observed BE in novices with still road photographs displayed for 5 seconds, it seems more plausible to suppose that BE in novices has not had sufficient time to develop. However, given that Munger et al. (2005) showed that BE is affected both by stimulus type (still photograph vs. approach sequence) and the individual differences in BE ratings with single photographs (i.e., baseline BE), this hypothesis must be treated with caution. The mechanisms affecting BE in approach sequences (e.g., priming effect as a result of the presentation of a wider-angle version of the to-be-memorised scene) deserve further research, including the field of expertise. Indeed, one limitation of the present research is that it did not include a baseline BE rating, as in Munger et al.’s study.

Thus, our findings suggest that expert knowledge modulates BE. The equivalent spatial layout extrapolation observed in beginners and experts suggests that drivers quickly become sensitive to the semantic information depicted by road scenes. The absence of directional distortion in novices leads us to question whether they extrapolate spatial layout at a later stage of scene perception, given that the scenes remained on screen only briefly (750 milliseconds). Indeed, using still road photos, Ménétrier and Didierjean (2013) observed BE in driving novices at a later stage of scene perception (5 seconds). Moreover, in the present experiment, novices verbally reported having perceived no difference between the last image of the approach sequence and the test photograph. This might be explained by the perceptual advantage classically observed in experts; that is, experts process the scenes depicting their field of expertise more effectively than novices (e.g., Reingold, Charness, Schultetus, et al., 2001). Analyzing the time course of BE may enable us to determine the evolution of the extrapolation process during scene perception as a function of expertise, a question that seems all the more relevant as BE occurs very quickly (e.g., Dickinson & Intraub, 2008).

In conclusion, this study suggests that expert knowledge is involved in BE. Indeed, BE was observed only in car drivers. The effect of expertise on BE suggests that the amount of knowledge available in LTM affects the spatial layout extrapolation process for scenes depicting the field of expertise of the observer at an early stage of scene perception. One question that arises from these observations, then, is whether BE requires more time to be deployed in nondrivers. Analyzing the time course of the phenomenon may help to answer this question. Moreover, an equivalent amount of BE was observed between beginners and experts, suggesting that the advantages of specific knowledge on the perception of scenes depicting the observer’s field of expertise emerge quickly. Such a mechanism seems adaptive in nature, enabling subjects to process the environment in which they are embedded more effectively.

## Note

1 The effect of ageing on BE has been the subject of only a few studies, which have led to a pattern of results that is not clear to date. Indeed, while Seamon et al.’s (2002) observations suggest that BE would be enhanced in older people compared with young adults, Multhaup, Munger, and Smith (2016) globally observed equivalent BE in young and older adults and, in some conditions, a tendency for less BE in the elderly.
